# Determinants of unintended pregnancy and induced abortion among adolescent women in Ethiopia: Evidence from multilevel mixed-effects decomposition analysis of 2000–2016 Ethiopian demographic and health survey data

**DOI:** 10.1371/journal.pone.0299245

**Published:** 2024-03-15

**Authors:** Tiruwork Amare, Fasil Tessema, Tamrat Shaweno

**Affiliations:** 1 MSI Ethiopia, Reproductive Choices, Addis Ababa, Ethiopia; 2 Jimma University Institute of Health Department of Epidemiology, Jimma, Ethiopia; 3 Africa Centres for Diseases Control and Prevention, Addis Ababa, Ethiopia; St. Paul’s Hospital Millennium Medical College, ETHIOPIA

## Abstract

**Background:**

Adolescents are highly at risk of unintended pregnancy due to physiological, sexual, social and psychological growth. The pregnancy may end with early childbirth, induced abortion and its complications. Although, the trends of unintended pregnancy and induced abortion have declined over time in Ethiopia, evidence is limited on key determinants for decline in order to propose vital areas of interventions. The current study aimed to identify the determinants of unintended pregnancy and induced abortion among adolescents over the decades.

**Methods:**

Trends in the prevalence of unintended pregnancy and induced abortion among adolescent women aged 15–19 years were investigated based using a series of the Ethiopia Demographic and Health Surveys (EDHS) data for the years 2000, 2005, 2011, and 2016. Sub-sample of adolescent women data was extracted from each survey. The combined datasets for unintended pregnancy and induced abortion over the study period (2000–2016) was analyzed. The percentage changes of trends of unintended pregnancy and induced abortion with its corresponding 95% CI for each variable were calculated. Multilevel mixed-effects decomposition analysis was applied to identify factors significantly associated with trends of unintended pregnancy and induced abortion among adolescents.

**Results:**

The trends of unintended pregnancy and induced abortion significantly declined during the study period. Unintended pregnancy among Ethiopian adolescents aged 15–19 years significantly decreased from 307 (41.4%) (95% CI: 35.7, 47.2%, p<0.001) in 2000 to 120 (25.1%) (95% CI: 18.9, 31.4%) in 2016. On the other hand, induced abortion significantly decreased from 62 (8.3%) (95% CI: 5.2, 11.4%) in 2000 to 20 (4.1%) (95% CI: 1.3, 6.9%, p = 0.004) in 2016. Age older than 18 years (Coeff = -0.41, 95%CI, -0.64, -0.18, p<0.001), living in Somali regional state (Coeff = -2.21, 95%CI, -3.27, -1.15, p<0.001) and exposure to media (Coeff = -0.60, 95%CI, -0.87, -0.33, p<0.001) showed a significance association with decline in unintended pregnancy whereas; living in Benshangul-Gumuz regional state (Coeff = -0.17, 95%CI, -0.32, -0.19, p = 0.03) and ANC service utilization history (Coeff = -0.81, 95%CI, -1.45, -0.17, p = 0.01) showed significance association with decline in induced abortion.

**Conclusion:**

The trends of unintended pregnancy and induced abortion significantly declined over the past decades in Ethiopia. Adolescent girls aged 17 years and above, exposure to media and living in Somali showed significant association with decline in unintended pregnancy whereas; living in Benshangul-Gumuz and ANC service utilization history showed significant decline with induced abortion. Exposure to media and utilization of Antenatal care (ANC) services may improve adolescent girls’ reproductive health uptake.

## Introduction

Unintended pregnancy is a pregnancy that is either unwanted, such as the pregnancy occurred when no children or no more children were desired [1]. Although, unintended pregnancy sharply fell from 1990–2014 globally, it didn’t show sharp declining trend in developing regions [2]. Globally, unintended pregnancy was 44% in 2014 and 65% of this burden happened in developing regions [3]. The magnitude of unintended pregnancy was highest in Africa and it was 24.0, 30.7, 35.9, 64.3% and 31.4 percent in Kenya, Egypt, Nigeria, South Africa and Ethiopia, respectively [3,4].

Induced abortion is the termination of a fetus that brought about intentionally using different options safe or unsafe based on quality of care [5,6]. Global estimates from 2010–2014 demonstrates that 45% of all induced abortions are unsafe and developing countries bear the burden of 97% of all unsafe abortions [5,6]. Abortion rates have declined significantly since 1990 in the developed world but not in the developing world [5]. In many areas of the world where rates of unintended pregnancy are high; unsafe abortions have also been shown to be high [7]. In Malawi, over 67% pregnancies are unintended and that of 27% ends with abortion [8]. Similarly, higher rate of abortion was reported in Nigeria [9] and in Congo [10].

According to World Health Organization (WHO)’s new study conducted in 36 countries, one in four pregnancies end up with unintended pregnancies and this was regarded as high [11]. Results from EDHS 2016 report indicate that unintended pregnancy and induced abortion are still high [12] and needs an ongoing priority intervention. Different study findings indicate that knowledge about contraceptive use, access to contraceptives, failure in contraceptive, sexual violence and level of decision on contraception choice can affect trends of unintended pregnancies and induced abortion among women. In addition, age and lower socio-economic status were also documented predictors of unintended pregnancies and induced abortions [2,3,6,8].

Information on trends of unintended pregnancy and induced abortion has a great role in developing strategies and prevention modalities to minimize complications emanating from unintended pregnancy and unsafe abortion. Although, there were studies conducted so far related to magnitude of unintended pregnancy and induced abortion among reproductive age women in Ethiopia [1–3], information is limited related to the determinants of trends of unintended pregnancy and induced abortion among adolescent women in Ethiopia. In addition, the magnitudes and determinants in different studies varied depending on the characteristics, study design and method of analysis. Moreover, information is limited on types of variables significantly playing role in decline or increment of unintended pregnancy and induced abortion among adolescents in Ethiopia. In areas with limited resource, identifying the key determinants of unintended pregnancy and induced abortion would be helpful for decision makers and policy analysts. Thus, this study aimed to determine the key predictors of unintended pregnancy, induced abortion and associated factors among adolescents in Ethiopia from 2000 to 2016.

## Methods

### Data source

The study utilized data from four consecutive Ethiopian Demographic and Health Surveys of 2000, 2005, 2011 and 2016 women’s data (IR data is the EDHS women’s data file code) set. According to the projected Ethiopian population report; by the year 2018 the estimated population of Ethiopia were 102.7 million of this 24.2% were adolescent and 11.7% of them were 15 to 19 year old. Adolescent girls were 5.8% from the total population [13]. The fertility rate was 56.6 births per 1000 women aged 15–19 years old. Until now, four demographic and health surveys were conducted in Ethiopia where reports are available in CSA online [12]. The first survey was conducted in 2000/1 followed by the 2005/6 EDHS which is part of the worldwide measure DHS project funded by USAID and was conducted under the auspices of the ministry of health and implemented by the then Population and Housing Census Commission Office (PHCCO). The EDHS surveys of 2011 and 2016 are also the same surveys like other years [12].

The EDHS used a two-stage stratified cluster sampling technique to select the study participants. In stage one, after each administrative region was stratified into urban and rural strata, Enumeration Areas (EAs) were selected using a probability proportional to EA size. In stage two, a household listing operation was carried out in all of the selected EAs and a fixed number of households from each EA were selected. All women aged 15–49 years who were permanent residents or who spend the night in the selected households the night before the survey were included [12]. In this analysis, a total weighted sample of 2,544 adolescent women (15–19 years old) who gave birth in the 5 years preceding the surveys or pregnant during the surveys or women who had unfavorable pregnancy outcomes were included. Trends in the prevalence of unintended pregnancy and abortion were investigated based on a series of the EDHS data for the years 2000 (n = 741), 2005 (n = 708), 2011 (n = 616), and 2016 (n = 479). Data from the surveys were appended together after extracting relevant variables for trend and multilevel mixed-effects decomposition analysis.

### Study variables

The dependent variables considered were trends of unintended pregnancy and induced abortion. Unintended pregnancy was defined as a pregnancy which is a sum of mistimed pregnancy (pregnancy wanted at a later time) and unwanted pregnancy (pregnancy which is not wanted at all) [3,7]. Abortion was taken from the EDHS question ‘have you ever had a pregnancy termination?’ with the response ‘yes’ if the woman ever had an abortion and otherwise ‘no’ as a binary outcome [12,14]. We used a simple Univariate filter, cross-tabulations, clinical knowledge and previous other related literature of interest; whether to decide which variables to keep in the analysis or not. The selected explanatory variables used in this analysis are indicated below [Table 1].

**Table 1:** List of selected variables for the decomposition analysis of trends of unintended pregnancy, induced abortion and associated factors among adolescents in Ethiopia using 2000–2016 EDHS data.

Unintended pregnancy		Induced abortion	
Socio demographic and economic variables	Reproductive history related variables	Socio-demographic and economic variables	Reproductive history related variables
Age, residence, educational status, religion, employment status, household wealth, region of residence, family size, household, decision exposure to media	ANC visit history, place of delivery, abortion, mode of delivery, birth order, pregnancy plan, knowledge about family planning, age at first sex, ever use of family planning	Age, residence, educational status, religion, employment status, household wealth, region of residence, family size, household, decision exposure to media	ANC visit history, place of delivery, mode of delivery, birth order, pregnancy plan, knowledge about family planning, age at first sex, ever use of family planning

### Data management and analysis

First, description of the study participants by socioeconomic, demographic and reproductive health services was made with frequencies and percent. This was followed by the estimation of the prevalence of unintended pregnancy and induced abortion by socioeconomic and reproductive health variables across survey years (2000, 2005, 2011 and 2016). Then, percentage point change with corresponding 95% CI of the outcome variables was calculated by each of the study factors including socio-economic, demographic and reproductive factors: to examine the changes over the survey years, 2000 to 2016. We used the combined dataset in order to detect any association between the study factors and the outcomes, as well as to examine trends in unintended pregnancy and abortion over the study years (2000–2016). Outcome variables with missing information (information missing on unintended pregnancy and abortion) were excluded from the study whereas; due to the cross sectional nature of the DHS survey, explanatory variables with greater than 5% missing value were excluded from further analysis. We estimated P for trends in each category of the study variables to assess whether the prevalence decreased or increased over the study period using chi-squared test for trend.

A multi-level mixed-effects decomposition logistic regression analysis of change in unintended pregnancy and induced abortion was applied to know the determinants for the changes in the trends of unintended pregnancy and induced abortion. The purpose of applying multilevel mixed-effects model was to take into account for the hierarchical nature of the DHS data whereas; application of decomposition analysis was to identify the sources of change in unintended pregnancy and induced abortion over the study periods. Bivariable multilevel mixed effects decomposition logistic regression analysis was carried to determine the coefficients at 95% confidence interval and those variables with p-value <0.25 were considered for multivariable decomposition analysis. In the multivariable multilevel mixed effects decomposition logistic regression analysis, those variables with p-value <0.05 were considered as significantly associated with outcome variable. The Intra-Class Correlation (ICC) was estimated to assess the cluster effect and the model fitness was compared using information criteria (IC). All statistical analyses were conducted using Stata version 18.0 with ‘svy’ command to adjust for sampling weights, clustering effects and stratification.

### Ethics statement and consent to participate

Ethical approval is not applicable for analysis and reporting from part of the EDHS data which is available upon reasonable request from the system. However, ethical clearance for the 2016 EDHS was provided by the Ethiopian Health and Nutrition Research Institute (EHNRI) Review Board, the National Research Ethics Review Committee (NRERC) at the Ministry of Science and Technology, the Institutional Review Board of ICF International, and the communicable disease control (CDC). We confirm that the study was conducted in accordance with the Helsinki Declaration. Written consent was obtained from each participant. In addition, parents/legal guardians were consented for minors aged 15 and 16 years. The parental consent for minors was also approved by the above mentioned national and international ethical bodies [12]. However, the dataset of the 2016 EDHS is not available as a public domain survey dataset. The author requested access to the data from demographic, health survey program team and access was granted to use the data for this research. Authorization letter was taken from http://www.measuredhs.com.

## Results

### Socio demographic and economic characteristics

In this study, a total of 2544 women age 15–19 years who gave birth in the 5 years preceding the surveys or pregnant during the surveys were included. About three-fourth of study participants were 18 and 19 years of age 1921 (75.5%) and the overall mean age was 18 years. Majority of respondents, 1547 (60.8%) had no formal education and 1148 (45.3%) of them were orthodox Christians. Around 1453 (60.7%) of participants were unemployed and 1395 (57.6%) of women were from poor household wealth status. Most, 2284 (89.8%) of women were married while only 1106 (43.5%) of them had media exposure (Table 2).

**Table 2:** Socio demographic and economic characteristics of adolescents in Ethiopia, 2000–2016.

Socio demographic and economic variables		Number	Percent
Age	15–17	623	24.5
	18–19	1921	75.5
Residence	Urban	228	8.9
	Rural	2316	91.1
Educational status	No education	1547	60.8
	Primary	871	34.2
	Secondary and higher	125	4.9
Marital status	Not married	260	10.2
	Married	2284	89.8
Religion	Orthodox	1148	45.3
	Protestant	389	15.3
	Muslim	946	37.3
	Others	54	2.1
Employment status	Not working	1453	60.7
	Working	940	39.3
Family size	2–3	1079	42.4
	4–5	846	33.3
	> = 6	619	24.3
Household wealth	Poor	1395	57.6
	Middle	694	28.6
	Rich	335	13.8
Regions category	Large central	2344	92.2
	Small peripheral	150	5.9
	Metropolis	50	1.9
Regions	Tigray	177	6.9
	Afar	31	1.2
	Amhara	678	26.7
	Oromia	1176	46.2
	Somali	74	2.9
	Benshangul-Gumuz	34	1.4
	SNNPR	313	12.3
	Gambella	11	0.4
	Harari	8	0.3
	Addis Ababa	33	1.3
	Dire Dawa	9	0.4
Media exposure	No	1438	56.5
	Yes	1106	43.5
Distance to health facility	Big problem	1283	71.2
	Not a big problem	521	28.8
Decision for health care	Not involved	1413	55.6
	Involved	1131	44.4

### Reproductive history of participants

About two-third 1719 (67.6%) had one live child and 718 (28.2%) had two or more children. Eight hundred ninety one (66.0%) were fifteen year old or younger during their first sex. Eight hundred twenty two (38.5%) of respondents had a history of at least one Antenatal care (ANC) visit, and only 278 (13.0%) delivered their index child in a health facility. Most, 2283 (89.8%) knew at least one family planning method while 342 (13.4%) correctly knew that a women is most likely to conceive half way between two periods and only 587 (23.1%) of them had ever used contraceptive. Moreover, 1677 (66.0%) had wanted (planned) the then pregnancies, 657 (25.9%) of them had wanted later (miss-timed) pregnancies while 206 (8.1%) did not want their pregnancies. On the other hand, 132 (5.2%) of respondents had abortion (Table 3).

**Table 3:** Reproductive history of adolescents in Ethiopia, 2000–2016.

Variables		Number	Percent
Parity	0	107	4.2
	1	1719	67.6
	2	609	23.9
	3–4	109	4.3
ANC visit history	No	1312	61.5
	Yes	822	38.5
Place of delivery	Home	1865	87.0
	Health facility	278	13.0
Mode of delivery	Non- Caesarean	2116	98.8
	Caesarean Section	26	1.2
Birth order	1^st^	2027	79.7
	2^nd^	447	17.6
	3^rd^ or 4^th^	70	2.7
Pregnancy plan status	Wanted then	1677	66.0
	Wanted later	657	25.9
	Not wanted at all	206	8.1
Knows any family planning	No	260	10.2
	Yes	2283	89.8
Ever use contraceptive	No	1957	76.9
	Yes	587	23.1
Knows ovulation period	Yes	342	13.4
	No	2201	86.6
Age at first sex(years)	≤ 15	891	66.0
	>15	460	34.0
Abortion experience	No	2412	94.8
	Yes	132	5.2

ANC: Antenatal care.

### Trends in unintended pregnancy among adolescents in Ethiopia

The magnitude of an unintended pregnancy among Ethiopian adolescents aged 15–19 years significantly decreased from 307 (41.4%) (95% CI: 35.7, 47.2%) in 2000 to 120 (25.1%) (95% CI: 18.9, 31.4%, p<0.001) in 2016.

Between the study period 2000 to 2016, the largest significant reduction in unintended pregnancy was observed among women with primary education (Diff = − 32.3; 95% CI: −48.4, −16.4), followed by those reside in metropolis regions (Diff = −29.0; 95% CI: −49.8, −8.3), those who had ever used contraceptive (Diff = −27.1; 95% CI: −43.4, −10.7) and whose age at first sex older than 15 years (Diff = −26.3; 95% CI: −44.3, −8.4). The magnitude of unintended pregnancy was also significantly reduced from 2000 to 2016 among women lived in Harari (Diff = −25.8; 95% CI: −46.7, −4.9), Tigray (Diff = −19.9; 95% CI: −36.5, −3.3) and Oromia (Diff = −13.6; 95% CI: −27.1, −0.2) regions. It was also significantly reduced among rural residents (Diff = −17.3; 95% CI: −26.3, −8.3), married women (Diff = −19.0; 95% CI: −27.6, −10.4), unemployed women (Diff = −23.5; 95% CI: −35.4, −11.5), women from poor household wealth (Diff = −20.0; 95% CI: −31.0,-9.0), women from male headed households (Diff = −20.9; 95% CI: −29.8, −12.1), women who had media exposure (Diff = −19.1; 95% CI: −34.3, −3.9) (Table 4).

**Table 4:** Trend of unintended pregnancy by socio demographic and economic variables among adolescent women in Ethiopia, 2000–2016.

Variables	2000 %(95%CI)	2005 %(95%CI)	2011 %(95%CI)	2016 %(95%CI)	2000–2016 n(%)	2016–2000 aDiff (95% CI)
**Age(years)**						
15–17	55.0(44.4–65.6)	45.7(33.8–57.6)	37.3(24.8-49-8)	34.1(21.8–46.4)	276(44.3)	-20.9(-37.2,-4.6)
18–19	36.9(30.5–43.3)	129.9(23.5–36.4)	30.7(24.0–37.3)	21.6(14.2–28.9)	588(30.6)	-15.3(-25.1,-5.6)*
**Residence**						
Urban	43.6(27.8–59.4)	40.8(23.5–58.1)	23.9(9.1–38.8)	38.7(15.9–61.5)	86 (37.6)	-4.9(-32.6, 22.8)
Rural	41.1(34.9–47.4)	33.4(27.1–39.7)	32.7(26.0–39.8)	23.8(17.4–30.3)	778(33.6)	-17.3(-26.3,-8.3)*
**Education**						
No education	36.6(30.2–43.1)	31.9(25.1–38.8)	25.4(17.3–33.5)	16.6(5.6–27.6)	479(31.0)	-20.0(-32.8,-7.2)*
Primary	60.0(46.4–73.8)	40.3(28.6–51.9)	39.1(30.5–47.6)	27.7(19.4–36.0)	330(37.9)	-32.3(, -48.4,-16.4)*
Secondary & higher	58.2(37.8–78.5)	27.8(0.9–54.7)	25.9(8.9–43.0)	40.0(18.1–62.0)	54(43.4)	-18.2(-48.1, 11.8)
**Marital status**						
Not married	55.9(40.8–76.1)	66.9(53.3–80.6)	61.7(39.7–83.7)	47.0(24.6–69.4)	148(57.2)	-8.9(-35.9,18.1)
Married	40.0(33.9–46.0)	30.9(24.8–37.0)	28,9(22.1–35.6)	21.0(15.0–27.1)	715(31.5)	-19.0(-27.6,-10.4)*
**Employment**						
Not employed	43.8(34.4–53.2)	32.0(25.3–38.7)	29.7(21.8–37.6)	20.3(13.0–27.6)	454(31.3)	-23.5(-35.4,-11.5)*
Employed	39.7(32.0–47.5)	41.3(30.5–52.1)	34.8(26.4–43.2)	32.3(20.7–43.8)	348(37.0)	-7.4(-21.4,6.4)
**Household wealth**						
Poor	41.1(34.5–47.6)	31.6(24.5–38.7)	26.4(17.5–35.2)	21.1(12.3–29.8)	443(31.8)	-20.0(-31.0,-9.0)*
Middle	43.1(31.9–54.3)	34.4(24.6–44.1)	37.5(23.3–51.8)	27.8(16.6–39.1)	251(36.3)	-15.3(-31.2,0.7)
Rich	35.3(8.3–62.2)	2.4(0.7–5.4)	37.4(27.8–46.9)	30.8(17.6–44.1)	112(33.5)	-4.5(-34.5, 25.7)
**Regions**						
Tigray	32.7(19.8–45.5)	8.2(1.8–14.6)	29.5(16.9–41.9)	12.8(2.3–23.2)	40(22.5)	-19.9(-36.5, -3.3)
Afar	21.3(9.7–32.8)	24.5(10.5–38.6)	13.0(2.2–23.8)	19.7(10.7–28.7)	6(20.1)	-1.6(-15.7, 12.7)
Amhara	50.3(40.7–59.8)	33.0(22.9–43.1)	35.5(20.8–50.2)	40.4(20.6–60.2)	277(40.9)	-9.9(-31.9.1,12.1)
Oromia	37.7(27.9–47.5)	38.5(14.8–33.4)	32.4(22.8–42.1)	24.1(14.8–33.4)	395(33.7)	-13.6(-27.1, -0.2)*
Somali	1.5(0.9–4.8)	6.1(2.7–14.9)	8.3(0.8–17.3)	0(0)	3(4.2)	-1.5(-4.8, 1.7)
Benshangul-Gumuz	35.6(22.2–48.9)	20.9(6.0–35.8)	31.0(21.3–40.7)	29.7(12.0–47.5)	10(29.9)	-5.9(-28.0,16.4)
SNNPR	38.0(20.7–55.3)	41.2(28.5–53.9)	29.1(13.9–44.2)	28.1(14.9–41.2)	108(34.4)	-9.9(-31.7, 11.8)
Gambella	40.0(21.6–58.3)	23.5(10.2–36.7)	43.3(27.2–59.4)	29.3(11.6–47.0)	4(35.4)	-10.7(-36.2,14.9)
Harari	33.8(14.4–53.1)	22.5(13.2–31.9)	39.1(19.9–58.4)	8.0(0.1–15.9)	2(25.8)	-25.8(-46.7,-4.9)*
Addis Ababa	60.5(39.4–81.5)	30.8(10.7–50.8)	67.3(41.6–92.9)	31.9(12.2–51.6)	16(47.1)	-28.6(-57.4, 0.3)
Dire Dawa	41.1(20.4–62.2)	11.1(0.3, 22.5)	30.6(11.3–49.9)	16.7(3.3–30.2)	2(23.5)	-24.4(-49.5, 0.3)
**Family size**						
2–3	40.8(32.4–49.3)	30.4(22.4–38.4)	30.2(21.5–38.9)	25.4(15.2–35.6)	340(32.1)	-15.4(-28.7, -2.2)*
4–5	44.7(35.2–54.1)	34.3(24.2–44.3)	23.5(14.2–32.7)	26.2(12.7–39.7)	283(33.4)	-18.5(-34.9, -2.1)*
>5	37.7(26.0–49.5)	40.9(28.3–53.5)	47.0(33.8–60.2)	23.9(14.5–33.4)	231(37.3)	-13.8(-28.9, 1.3)
Media exposure						
No	36.5(30.0–42.9)	32.8(25.9–39.5)	28.8(17.6–40.0)	21.1(13.3–29.0)	444(30.9)	-15.4(-25.5,-5.2)*
Yes	51.0(40.8–61.2)	35.6(25.0–46.3)	33.8(26.5–41.2)	31.9(20.7–43.1)	419(37.9)	-19.1(-34.3,-3.9)
Overall	41.4(35.7–47.2)	33.9(27.9–39.9)	31.9(25.7–38.3)	25.1(18.9–31.4)	863(34.0)	-16.3 (-24.8, -7.8)*

*CI: Confidence Interval, aDiff: Percentage change in prevalence of unintended pregnancy between 2000 to 2016, SNNPR: South Nations Nationalities and Peoples Region, *: Significant for trend at p-value <0.05.

In addition, significant decline of unintended pregnancy was detected among women with home delivery (Diff = −18.0; 95% CI: −29.7, −6.3), women who had no abortion (Diff = −18.4; 95% CI: −27.1, −9.7) and those who had ANC history (Diff = −23.9; 95% CI: −36.8, −11.0). Similarly, it was significantly reduced among women from 2 to 3 family size (Diff = −15.4; 95% CI: −28.7, −2.2), from 4–5 family size (Diff = −18.5; 95% CI: −34.9, −2.1), women who had one live child (Diff = −14.9; 95% CI: −24.8, −4.9) and women who had 2–4 live children (Diff = −22.9; 95% CI: −41.2, −4.7) (Table 5).

**Table 5:** Trend of unintended pregnancy by reproductive variables among adolescent women in Ethiopia, EDHS 2000–2016.

Variables	2000 n (%)	2005 n (%)	2011 n (%)	2016 n (%)	2000–2016 n(%)	2016–2000 aDiff (95% CI)
Parity						
0	31.7(12.6–50.7)	8.4(6.7–23.5)	53.4(11.1–95.8)	38.5(8.5–85.5)	31(28.9)	6.8(-4.4, 57.8)
1	38.8(31.9–45.7)	36.0(28.9–43.1)	29.4(23.2–35.6)	24.0(16.8–31.1)	562(32.7)	-14.9(-24.8, -4.9)*
2–4	50.0(39.3–60.7)	33.7(21.5–45.9)	35.9(23.8–47.9)	27.1(12.3–41.8)	270(37.7)	-22.9(-41.2, -4.7)*
Sex of HH Head						
Male	41.6(35.7–47.5)	33.6(27.3–40.0)	31.6(24.5–38.8)	20.6(14.1–27.2)	729(33.3)	-20.9(-29.8,-12.1)*
Female	40.0(24.2–55.8)	36.3(22.4–50.1)	33.6(21.0–46.1)	45.4(31.6–59.2)	134(38.5)	5.4(-15.5, 26.4)
Place of delivery						
Home	44.4(37.7–51.1)	33.6(26.9–40.3)	35.2(27.4–43.1)	26.4(16.9–35.9)	676(36.3)	-18.0(-29.7,-6.3)*
Health facility	32.0(13.9–50.2)	43.3(23.0–63.5)	18.1(11.7–35.1)	24.9(14.5–35.5)	75(26.9)	-7.1(-28.0, 13.9)
Ever use FP						
No	39.4(33.2–45.5)	34.8(28.7–40.9)	31.5(23.5–39.6)	24.8(17.2–32.3)	666(34.1)	-14.6(-24.3,-4.9)*
Yes	52.7(39.5–65.8)	28.1(14.0–42.2)	33.0(24.3–41.7)	25.6(15.9–35.4)	197(33.6)	-27.1(-43.4,-10.7)*
Ovulation period						
Knows	44.6(29.7–59.5)	32.3(17.7–46.9)	26.6(10.9–42.4)	31.1(16.1–45.9)	114(33.5)	-13.5(-34.7,7.5)
Don’t know	41.0(34.9–47.2)	34.1(27.5–40.8)	32.9(26.6–39.4)	23.8(16.7–30.8)	749(34.1)	-17.2(-26.7,-7.8)*
Age at first sex						
< = 15	46.5(36.2–56.9)	38.2(29.9–46.5)	43.4(28.7–58.1)	28.9(20.1–37.7)	339(38.1)	-17.6(-31.3, -4.0)
>15	46.2(30.1–61.4)	37.8(24.1–51.4)	41.8(20.9–62.7)	19.9(10.2–29.5)	147(32.0)	-26.3(-44.3, -8.4)*
Abortion						
No	43.5(37.5–49.5)	35.0(28.9–41.1)	32.9(26.5–39.3)	25.1(18.9–31.3)	843(34.9)	-18.4(-27.1,-9.7)*
Yes	18.7(5.7–31.8)	0(0)	13.4(3.1–29.9)	26.1(12.9–65.1)	20(15.5)	7.4(-33.9,48.5)
ANC history						
No	41.8(34.3–49.4)	35.8(28.8–42.9)	34.2(25.2–43.1)	31.3(15.0–47.7)	487(37.1)	-10.5(-285,7.6)
Yes	47.9(37.1–58.7)	28.6(16.5–40.6)	32.9(22.5–43.3)	24.0(17.1–30.9)	262(21.9)	-23.9(-36.8,-11.0)*

*CI: Confidence Interval, aDiff: Percentage change in prevalence of unintended pregnancy between 2000 to 2016, HH: House Hold, FP: Family Planning, ANC: Ante Natal Care, *: Significant for trend at p-value <0.05.

### Trends of abortion among adolescent women in Ethiopia

The prevalence of abortion significantly decreased from 62 (8.3%) (95% CI: 5.2, 11.4%) in 2000 to 20 (4.1%) (95% CI: 1.3, 6.9%, p = 0.004) in 2016. Significant reduction in percentage point of abortion was observed among women with intended pregnancy (Diff = −7.5; 95% CI: −12.9, −2.1), women who had no media exposure (Diff = −6.7; 95% CI: −12.3, −1.2), women who had one live child (Diff = −6.1; 95% CI:−10.9−1.2) and women resided in large central regions (Diff = −4.5; 95% CI: −9.1,−0.1) (Table 6).

**Table 6:** Trends of induced abortion by variables among adolescent women in Ethiopia, EDHS 2000–2016.

Variables	2000 %(95%CI)	2005 %(95%CI)	2011 %(95%CI)	2016 %(95%CI)	2000–2016 n(%)	2016–2000 aDiff% (95% CI)
**Age(years)**						
15–17	4.6(0.4–8.8)	2.0(0.4–4.4)	3.9(1.4–9.2)	3.1(1.6–7.8)	21(3.4)	-1.5(-7.8,4.8)
18–19	9.5(5.7–13.3)	3.6(0.6–6.6)	4.7(1.9–7.5)	4.4(0.8–8.1)	111(5.7)	-5.0(-10.3,0.3)
**Residence**						
Urban	5.9(1.5–13.3)	11.2(0.7–21.7)	11.1(1.4–23.7)	1.2(0.7–3.1)	17(7.4)	-4.7(-12.4,3.1)
Rural	8.6(5.2–11.9)	2.6(0.2–4.9)	3.9(1.5–6.4)	4.3(1.3–7.4)	115(4.9)	-4.2(-8.8(0.3)
**Education**						
No education	8.1(5.2,12.3)	4.0(1.8,8.7)	4.3(2.1,8.8)	6.6(2.1,19.5]	91(5.8)	-1.4(-9.6,6.8)
Primary and above	8.0(4.2,18.4)	1.1(0.3,3.5)	4.7(2.0,10.8)	2.9(1.2,6.9)	41(4.1)	-6.1(-13.3,1.1)
**Employment**						
Not employed	8.3(4.3,15.6)	3.2(1.3,7.5)	4.6(2.1,9.8)]	3.3(1.3,8.3)	65(4.5)	-5.0(-11.3,1.3)
Employed	8.5(4.7,14.9)	3.3(1.1,9.9)	3.9(1.7,8.0)	5.3(1.8,14.4)	54(5.7)	-3.3(-10.7,4.1)
**Household wealth**						
Poor	9.9(6.5,14.6)	4.3(1.6,10.6)	3.8(1.8,7.9)	6.4(1.4,11.4)	89(6.4)	-3.4(-9.8,2.9)
Middle	2.6[0.5,13.4)	1.1(0.2,5.2)	5.7(1.8,16.9)	2.6(1.3,6.5)	19(2.7)	-0.1(-5.9,5.9)
Rich	14.1(2.8,48.7)	29.4(10,61.0)	5.0(1.9,13.2)	0.6(0.4–1.6)	17(4.9)	-13.5(-34.8,7.7)
**Regions category**						
Large central	8.5(5.7,12.4)	3.0(1.3,6.8)	4.4[2.4,7.9]	4.0(1.8,8.6)	121(5.2)	-4.5(-9.1,-0.1)
Small peripheral	4.1(1.7,9.7)	4.1[0.5,25.1]	3.6(0.7,15.4)	5.8(2.7,12.2)	7(4.4)	1.7(-3.9,7.3)
Metropolis	6.3(2.1,17.4)	8.7(2.8,23.6)	18.4(4.3,53.2)	2.2(0.8,6.5)	4(8.2)	-4.1(-11.1,3.0)
**Family size**						
2–3	11.5(6.3–16.7)	4.3(1.0–7.7)	4.8(1.0–8.6)	5.9(0.4–11.9)	73(6.9)	-5.5(-13.4,2.4)
4–5	5.2(0.8–9.7)	1.0(0.8–9.7)	3.5(0.1–7.1)	3.9(1.5–9.3)	29(3.3)	-1.4(-8.4,5.6)
>5	6.8(0.6–14.2)	4.8(3.7–13.2)	5.6(0.4–11.6)	1.7(1.0–4.4)	30(4.8)	-5.1(-12.9,2.8)
**Parity**						
0	10.7(3.3–24.8)	17.6(3.1–38.1)	3.2(0.4–9.9)	0.8(0.1–2.7)	11(10.2)	-9.9(-24.1,4.2)
1	9.7(5.9–13.6)	2.4(0.6–4.2)	6.1(2.7–9.4)	3.6(0.6–6.7)	98(5.7)	-6.1(-10.9,-1.2)*
2–4	4.1(1.5–9.7)	2.7(2.4–7.7)	1.1(0.4–2.5)	5.9(2.8–14.5)	23(3.2)	1.8(-8.5,12.1)
**Sex of HH Head**						
Male	8.6(5.2–11.9)	3.5(0.9–6.1)	5.6(2.5–8.7)	4.9(1.4–8.4)	127(5.8)	-3.6(-8.5,1.2)
Female	5.7(1.8–13.2)	0.7(0.6–1.9)	0.01(0.02–0.07)	0.2(0.1–0.6)	5(1.4)	-5.5(-12.9,2.1)
**Media exposure**						
No	10.2(6.0–14.4)	3.4(0.1–6.9)	5.8(0.9–10.8)	3.5(0.1–7.1)	88(6.1)	-6.7(-12.3,-1.2)*
Yes	4.5(0.5–8.5)	2.8(0.3–5.4)	3.8(1.2–6.5)	5.1(0.2–10.3)	44(3.9)	0.6(-5.9,7.2)
**Place of delivery**						
Home	7.9(4.4–11.5)	3.3(0.5–6.1)	3.4(0.9–5.9)	5.0(0.3–9.7)	93(4.9)	-2.9(-8.8,2.9)
Health facility	18.2(0.3–36.2)	11.2(2.0–24.3)	11.2(3.8–26.3)	1.8(0.9–4.5)	21(7.4)	-16.5(-34.7,1.7)
**Ever use FP**						
No	8.6(5.2–12.0)	3.0(0.4–5.6)	4.5(1.5–7.5)	3.9(0.7–7.2)	103(5.3)	-4.6(-9.4,0.1)
Yes	6.5(0.4–13.4)	4.4(0.6–9.3)	4.7(0.2–9.2)	4.2(1.1–9.5)	29(4.8)	-2.3(-11.0,6.4)
**Pregnancy plan**						
Intended	11.5(6.9–16.1)	4.9(1.4–8.3)	5.8(2.4–9.2)	4.0(1.2–6.8)	111(6.6)	-7.5(-12.9,-2.1)*
Unintended	3.7(0.9–6.6)	0	1.9(1.0.5–4.3)	4.2(3.4–11.9)	21(2.4)	0.5(-7.7,8.7)
**ANC history**						
No	8.1(3.9–12.2)	4.6(1.0–8.2))	3.2(0.7–5.7)	5.4(3.7–14.5)	72(5.5)	-2.7(-12.7,7.3)
Yes	10.9(3.3–18.6)	1.0(0.5–2.4)	5.7(0.4–10.9)	3.2(0.4–6.0)	41(4.9)	-7.7(-15.9,0.4)
Overall	8.3(5.2–11.4)	3.2(0.9–5.5)	4.6(2.1–7.0)	4.1(1.3–6.9)	5.2(3.9–6.7)	-4.2 (-8.4–0.03)*

*CI: Confidence Interval, aDiff: Percentage change in prevalence of unintended pregnancy between 2000 to 2016, HH: House Hold, FP: Family Planning, ANC: Ante Natal Care, *: Significant for trend at p <0.05.

### Decomposition analysis for unintended pregnancy and abortion

After controlling the role of changes in compositional characteristics, age (Coeff = -0.41, 95%CI, -0.64, -0.18, p<0.001) and living in Somali regional state (Coeff = -2.21, 95%CI, -3.27, -1.15, p<0.001) showed a significance association with decline in unintended pregnancy whereas; living in Benshangul-Gumuz regional state (Coeff = -0.17, 95%CI, -0.32, -0.19, p = 0.03) showed significance association with decline in abortion from socio demographic and economic variables (Table 7).

**Table 7:** Decomposition analysis for trends of unintended pregnancy and abortion by socio demographic and economic variables among adolescent women in Ethiopia, 2000–2016.

Variables	Unintended pregnancy			Abortion		
	Coeff.	P-value	95%CI	Coeff.	P-value	95%CI
**Age(years)**						
15–17	Ref			Ref		
18–19	-0.41	<0.001	-0.64, -0.18	0.41	0.12	-0.11, 0.34
**Residence**						
Urban	Ref			Ref		
Rural	-0.42	0.09	-0.69, 0.05	0.81	0.025	0.10, 1.51
**Education**						
No education	Ref			Ref		
Primary	0.31	0.005	0.09, 0.53	-0.10	0.67	-0.56, 0.36
Secondary & higher	-0.03	0.89	-0.47, 0.41	-0.22	0.62	-1.10, 0.66
**Marital status**						
Not married	Ref					
Married	1.08	<0.001	0.70. 1.70	-0.33	0.49	-1.32, 0.62
**Employment**						
Not employed	Ref			Ref		
Employed	-0.05	0.82	-0.48, 0.37.8	-0.22	0.64	-1.18, 0.73
**Household Wealth**						
Poor	Ref			Ref		
Middle	0.12	0.49	-0.22, 0.48.0	0.61	0.12	-1.14, 1.36
Rich	0.22	0.20	-0.11, 0.56	0.22	0.19	-0.11, 0.56
**Regions**						
Tigray	0.26	<0.001	-0.21, 0.74	-0.03	0.96	-0.93, 0.88
Afar	0.01	0.96	-0.62, 0.66	0.73	0.24	-1.11, 1.21
Amhara	1.06	<0.001	0.63, 1.52	0.32	0.42	-0.46, 1.11
Oromia	0.8	0.23	0.32, 0.94	0.19	0.64	-0.60, 0.98
Somali	-2.21	<0.001	-3.27, -1.15	0.23	0.60	-0.64, 1.10
Benshangul-Gumuz	0.59	0.014	0.12, 1.27	-0.17	0.03	-0.32, -0.19
SNNPR	0.86	0.001	0.37, 1.36	-0.25	0.64	-1.30, 0.81
Gambella	0.74	0.03	0.25, 1.25	0.11	0.84	-0.89, 1.11
Harari	0.33	0.21	-0.19, 0.87	0.45	0.34	-0.47, 1.38
Dire Dawa	0.02	0.96	-0.62, 0.66	0.05	0.93	-1.11, 1.21
Addis Ababa	Ref			Ref		
**Family size**						
2–3	Ref			Ref		
4–5	-0.18	0.18	-0.45, 0.08	0.52	0.07	-0.04, 1.08
>5	0.03	0.86	-0.25, 0.29	-0.14	0.66	-0.78, 0.49

*CI: Confidence Interval, SNNPR: South Nations Nationalities and Peoples Region, HH: House Hold, FP: Family Planning, ANC: Ante Natal Care.

From a total of nine reproductive history related variables entered into a multilevel mixed effect decomposition logistic regression model, an exposure to media (Coeff = -0.60, 95%CI, -0.87, -0.33, *p*<0.001) showed a significance association with decline in unintended pregnancy. Similarly, from eight reproductive health related variables entered into final multilevel mixed effect decomposition logistic regression model; ANC history (Coeff = -0.81, 95%CI, -1.45, -0.17, *p* = 0.01) was significantly associated with induced abortion decline from 2000–2016 (Table 8).

**Table 8:** Decomposition analysis for trends of unintended pregnancy and abortion by reproductive history variables among adolescent women in Ethiopia, 2000–2016.

Variables	Unintended Pregnancy			Abortion		
	Coeff.	p-value	95%CI	Coeff.	p-value	95%CI
Parity						
0	Ref			Ref		
1	0.37	0.24	-0.23, 0.98	0.04	0.96	-1.43, 1.51
2–4	0.35	0.25	-0.28, 0.98	0.26	0.73	-1.24, 1.77
Sex of HH Head						
Male	Ref			Ref		
Female	0.04	0.82	-0.27, 0.34	0.93	0.05	-0.01, 1.88
Media exposure						
No	Ref					
Yes	-0.60	<0.001	-0.87, -0.33	0.36	0.29	-0.30, 1.01
Place of delivery						
Home	Ref			Ref		
Health facility	0.35	0.043	0.007, 0.71	0.58	0.16	-0.23, 1.40
Ever use FP						
No	Ref			Ref		
Yes	0.21	0.19	-0.10, 0.53	0.06	0.88	-0.66, 0.77
Ovulation period						
Knows	-0.11	0.55	-0.47, 0.25	0.18	0.70	-0.71, 1.07
Don’t know	Ref			Ref		
Age at first sex						
< = 15	Ref					
>15	-0.04	0.76	-0.33, 0.24	0.31	0.39	-0.39, 1.02
Abortion						
No	Ref					
Yes	0.76	0.047	-1.50, -0.01			
ANC history						
No	Ref			Ref		
Yes	0.18	0.23	-0.11, 0.47	-0.81	0.01	-1.45, -0.17

*CI: Confidence Interval, HH: House Hold, FP: Family Planning, ANC: Ante Natal Care.

## Discussion

We assessed the determining factors for trends of unintended pregnancy and abortion among adolescent women in Ethiopia for the years and we found that there was a significant overall decline. Although, this finding was in line with similar other studies [4,15–19], unintended pregnancy rate in Ethiopia still remains high. The significant decline of unintended pregnancy may be due to the impacts of the national reproductive health interventions that were effectively implemented in Ethiopia [12–15]. Factors associated with significant decline of unintended pregnancy during the past decades included; adolescent women being aged above 17 years and adolescent women who used to live in Somali regional state.

Similarly, the trend of induced abortion significantly declined from 8.3% in 2000 to 4.1% in 2016 in Ethiopia and still the magnitude of abortion is high. This figure is consistent when compared to similar other studies [6–8]. This significant reduction in an induced abortion may be attributed to the relatively increased maternal health service utilization like modern contraceptive use by the currently married women (35%)) in 2016 [5,12] compared to (6%) in 2000 [12,17,20].

The trend of unintended pregnancy significantly declined among adolescent women aged 18 years and above compared to adolescent women aged below 18 years. Studies conducted in developing countries indicated the risk of unplanned pregnancies decreases with relatively older age [21–26]. This might be due to older women had relatively better knowledge on contraceptive methods to prevent unintended pregnancy and lower contraceptive failure rate [21,24]. Moreover, as they are getting older, women might also become more literate about the importance and accessibility of reproductive or maternal health services. In addition, this could be also as the results of older women are less likely to engage in risky sexual behaviors such as unprotected sexual intercourse and sex under the influence of drinking alcohol [25,26]. However, other literatures revealed a negative relationship between age and unintended pregnancy [23,27,28]. This finding might be related to the fact that adult women might already have the desirable number of children and considered any additional pregnancy as mistimed or unwanted.

The current finding also suggests that the trend of unintended pregnancy significantly reduced among adolescent women residing in Somali regional state of Ethiopia. Evidences indicated that in some Ethiopian regions including Afar, Benshangul-Gumuz, Somali, and Oromia; half or more of all girls are married before age 18 [29]. Child marriage is significantly associated with a history of rapid repeat childbirth and not using contraception before first childbirth; which leads to the pregnancy to be wanted [30]. Thus, the significant decrease in unintended pregnancy among adolescent women in Somali region may be due to the increased child marriage that may end with intended pregnancy [29,30–34]. Furthermore, Somali and Afar regions have the lowest proportion of women and men with desire to limit childbearing and have the highest total wanted fertility rate in Ethiopia compared to the other regions [32]. The lowest average of contraceptive use rate (8.8%) in Ethiopia in 2021 was recorded in Somali region [33].

Induced abortion significantly declined among adolescent women with ANC history. This finding is consistent with studies conducted in Kenya [25], in Ethiopia [35–39], in South Africa [40] and in United States [41]; reported that previous exposure to ANC had influenced induced abortion. This might be explained as women having a previous exposure to ANC follow-up and have given birth might be influenced to have an additional baby rather than planning for abortion [24–27].

Similarly, induced abortion significantly declined among adolescent women residing in Benshangul-Gumuz regional state of Ethiopia during the study period. This may be explained from different angles. The abortion rate continued to be lowest (6.7 per 1,000 in the least densely populated and most traditional rural regions (Afar, Benshangul-Gumuz, Gambella and Somali), apparently for the reason that of limited access to services, lower utilization of abortion services or both [42]. Moreover, abortion in Somalia is legal only to save the pregnant person’s life [8].

### Strengths and limitations of the study

The research is based on nationally representative data with large sample size so that the observed findings more likely to show the trends of unintended pregnancy and abortion among adolescent women in Ethiopia. It has the potential to provide evidence for policy-makers and program planners to design appropriate intervention strategies both at national and regional levels because the estimates are based on the national survey data. Nevertheless, the study had limitations because of the Ethiopian demographic and health surveys data are mostly based on respondents’ self-report and could have the possibility of recall bias. In addition, it is difficult to establish a cause-effect relationship between the outcomes and independent variables due to the research is based on cross-sectional data. Moreover, the precision of trend analysis by regions in Ethiopia may be questioned as the distribution of unintended pregnancy and induced abortion among adolescent women across some regions in Ethiopia is small and may give wrong impressions for readers. Hence, the findings need to be interpreted with caution.

## Conclusion

The trends for both unintended pregnancy and induced abortion significantly declined over the study period. Despite this reduction, unintended pregnancies and induced abortion are high in Ethiopia. The significant decline in unintended pregnancy among adolescent women was observed among adolescent women aged older than 18 years, living in Somali regional state and exposure to media whereas; the significant decline in induced abortion is observed among adolescent women living in Benshangul-Gumuz regional state and having previous ANC service utilization history. Intervention strategies including expansion of an exposure to media to increase their knowledge and special attention to regions with high child marriage in Ethiopia might decrease unintended pregnancy and induced abortion among adolescent women. Moreover, scaling up of MCH services including ANC services can keep the momentum of decline in an induced abortion.

## Abbreviations

CAC: Comprehensive Abortion Care
CSA: Central Statistics Agency
DHS: Demographic and Health Survey
EDHS: Ethiopia Demographic and Health Survey
HAPCO: HIV/AIDS Prevention and Control Office
MDG: Millennium Development Goals
PHCCO: Population and Housing Census commission Office
SRH: Sexual and Reproductive Health
